# Function and therapeutic prospects of next-generation probiotic *Akkermansia muciniphila* in infectious diseases

**DOI:** 10.3389/fmicb.2024.1354447

**Published:** 2024-02-06

**Authors:** Lifeng Li, Mingchao Li, Yihua Chen, Zengyuan Yu, Ping Cheng, Zhidan Yu, Weyland Cheng, Wancun Zhang, Zhaobao Wang, Xueyan Gao, Huiqing Sun, Xiaolei Wang

**Affiliations:** ^1^Henan International Joint Laboratory of Children’s Infectious Diseases, Department of Neonatology, Children’s Hospital Affiliated to Zhengzhou University, Henan Children’s Hospital, Zhengzhou Children’s Hospital, Zhengzhou, China; ^2^Electrical Biology Room, Children’s Hospital Affiliated to Zhengzhou University, Henan Children’s Hospital, Zhengzhou Children’s Hospital, Zhengzhou, China; ^3^Energy-rich Compounds Production by Photosynthetic Carbon Fixation Research Center, Shandong Key Lab of Applied Mycology, College of Life Sciences, Qingdao Agricultural University, Qingdao, China; ^4^Medical Science and Technology Innovation Center, Shandong First Medical University and Shandong Academy of Medical Sciences, Jinan, China; ^5^State Key Laboratory Cultivation Base, Shandong Provincial Key Laboratory of Ophthalmology, Eye Institute of Shandong First Medical University, Qingdao, China

**Keywords:** next-generation probiotics, *Akkermansia muciniphila*, infectious diseases, intestinal flora, immune regulation

## Abstract

*Akkermansia muciniphila* is a gram-negative bacterium that colonizes the human gut, making up 3–5% of the human microbiome. *A. muciniphila* is a promising next-generation probiotic with clinical application prospects. Emerging studies have reported various beneficial effects of *A. muciniphila* including anti-cancer, delaying aging, reducing inflammation, improving immune function, regulating nervous system function, whereas knowledge on its roles and mechanism in infectious disease is currently unclear. In this review, we summarized the basic characteristics, genome and phenotype diversity, the influence of *A. muciniphila* and its derived components on infectious diseases, such as sepsis, virus infection, enteric infection, periodontitis and foodborne pathogen induced infections. We also provided updates on mechanisms how *A. muciniphila* protects intestinal barrier integrity and modulate host immune response. In summary, we believe that *A. muciniphila* is a promising therapeutic probiotic that may be applied for the treatment of a variety of infectious diseases.

## 1 Introduction

The surface of human oral-gastrointestinal tract resides more than 100 trillion microorganisms including bacteria, fungi, parasites and viruses (Kamada et al., 2013). Researchers have found that gut microbiota has a complex and close relationship with human health and disease. Gut microbiota play critical roles in host immune regulation, inflammatory response, and energy metabolism. The disturbances or imbalances of gut microbiota are related to the development of a variety of diseases, such as inflammatory bowel disease (IBD), metabolic syndrome, obesity, diabetes, and inflammation (Biedermann and Rogler, 2015). At present, there are more and more adverse reactions caused by the abuse of antibiotics, and the use of probiotics can reduce these adverse reactions, which brings new hope for human treatment and improvement of diseases. Probiotics currently used are several organisms conferring health benefit for the host.

In recent years, with the development of gut microbiome sequencing and strain isolation technology, new strains with potential health benefits were gradually found with no human applications, which are called the Next-generation probiotics (NGPs) (O’Toole et al., 2017). The development of NGPs is more likely aiming for pharmaceutical use than a food delivery route; hence, it can also be termed live biotherapeutic products (LBPs). Hence, there are some differences in the history and route to market between probiotics and NGPs.

**Figure 1:**
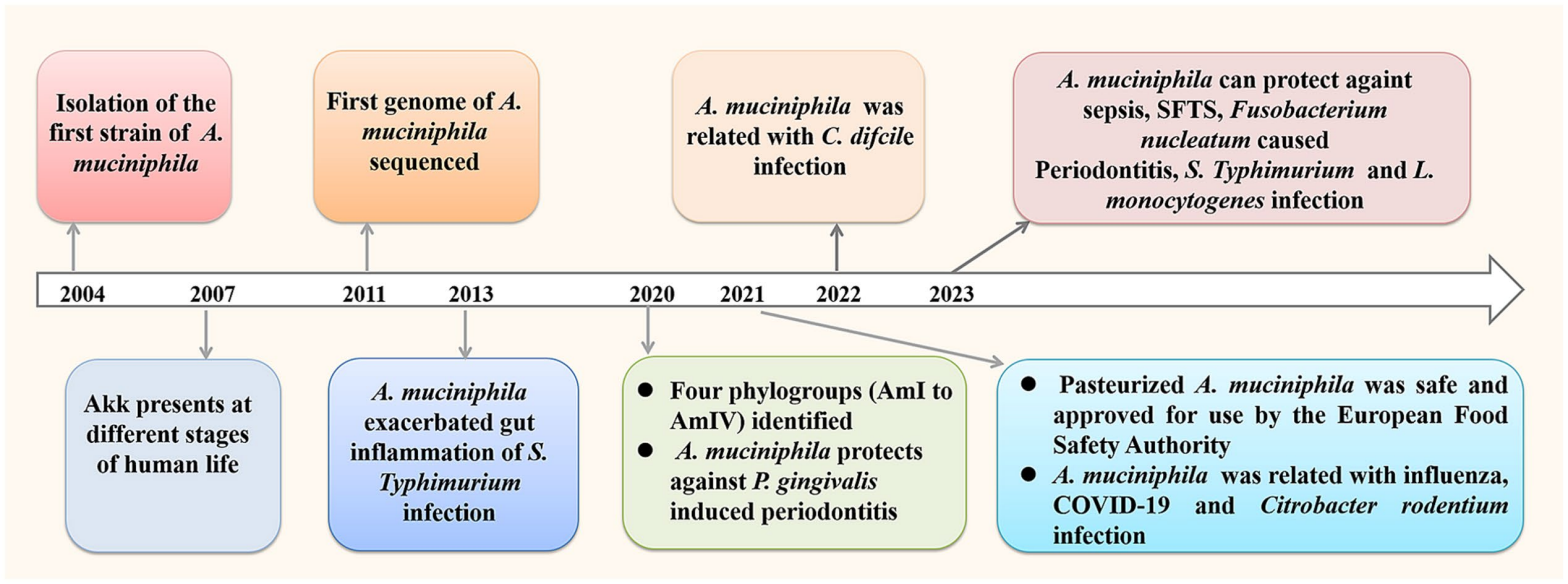
Research process of *A. muciniphila* with a focus on infectious diseases.

*Akkermansia muciniphila* (*A. muciniphila*) is a next- generation probiotics with promising clinical application prospects, which is a resident of the human gut, making up 3–5% of the human microbiome (Derrien et al., 2004). It can grow in the intestinal mucus layer and feed on mucin secreted by the host, thereby colonizing the intestine through competitive rejection and protecting the intestine from pathogens. According to recent studies, *A. muciniphila* has shown beneficial effects on various fields including anti-cancer, delaying aging, reducing inflammation, improving immune function, regulating nervous system function, etc. Studies have also shown that oral doses of l × 10^10^ colony forming unit (CFU) in human volunteers are very safe, regardless of whether *A. muciniphila* bacteria are live or dead (Plovier et al., 2017). In 2021, the European Food Safety Authority confirmed the safety of pasteurized *A. muciniphila* and approved it for use as a novel food pursuant (EFSA et al., 2021). There is growing interest on the research of *A. muciniphila*, and many animal experiments confirm its positive roles in infectious diseases (Figure 1).

In this review, we provided a brief summary of the process on the basic characteristics and diversity of *A. muciniphila*, the impact and action mechanism of *A. muciniphila* and its derived components on infectious diseases, such as sepsis, virus infection, enteric infection and periodontitis. We also summarize the interaction mechanisms between *A. muciniphila* and host, focusing on the protection of intestinal barrier integrity and regulation of immune response and host metabolism.

## 2 Overview of *Akkermansia muciniphila*

Muriel Derrien et al. isolated a mucin-degrading bacterium from human feces and named it *Akkermansia muciniphila* in 2004, which is represented by the typical strain Muc^T^ (ATCC BAA-835) (Derrien et al., 2004). *A. muciniphila* is anaerobic and currently the only known member of the human intestinal *Vermicillae* residents. The cells of the Muc^T^ strain were oval-shaped, and the cell size varied with the culture medium. In the Muc^T^ medium, the diameter of strain Muc^T^ was 640 nm and the length was 690 nm. In BHI medium, the diameter of Muc^T^ strain was 830 nm and the length was 1 μm. Electron microscope shows filamentous structures in the surface of the bacteria growing in mucous media, which are capsular polymers used to connect cells and may contribute to bacterial adhesion and colonization in the gastrointestinal tract. The bacteria can grow at 20–40°C and pH 5.5–8.0, the optimum growth was at 37°C and pH 6.5 using mucin as sole carbon and nitrogen resource. Initial report indicated *A. muciniphila* is strictly anaerobic, whereas Ouwerkerk et al. reported that *A. muciniphila* is oxygen tolerant (Ouwerkerk et al., 2016). *A. muciniphila* can survive at nanomolar levels of oxygen, and the oxygen induced a complex transcriptional response of the bacteria by. *A. muciniphila* can survive for 48 h in ambient air, with a 25% survival rate after 24 h and with only 1% survival rate after 48 h.

*A. muciniphila* is present in distinct parts of the human mucosa and fecal samples based on 16S rRNA gene sequencing (Eckburg et al., 2005). It was also isolated from blood-culture sample of an 18-year-old woman (Dubourg et al., 2017), and the breast milk of healthy human (Hou et al., 2023). *A. muciniphila* presents at different stages of human life, whereas the levels varied between different ages and regions. *A. muciniphila* colonization starts in early life and increases to a level similar to adults (10^8^ cells/g) within a year, whereas the abundance of the elderly decreased (Collado et al., 2007). Derrien et al. reported an abundance of *A. muciniphila* over 1% (Derrien et al., 2008). Guo et al. reported that the colonization rate was 51.74% among the sample populationfrom southern China (Guo et al., 2016). *A. muciniphila* colonization rate in southern China was 51.71%, which was 74.70%in European populations. Considering influences of different regions, living environment and diet structure on microbial colonization, and further analysis of the correlation between age and *A. muciniphila* abundance is needed.

Mucin is the main component of human intestinal epithelium (Liévin-Le Moal and Servin, 2006). *A. muciniphila* can grow in the intestinal mucus layer and “feed” on mucin secreted by the host, and colonize in the intestinal tract through competitive rejection and protect the intestinal tract from pathogens. Although *A. muciniphila* uses mucin as an energy source, numerous observations have confirmed that *A. muciniphila* played positive regulatory function on the thickness and stability of intestinal mucus layer and the integrity of intestinal barrier.

## 3 Genome diversity and properties of *Akkermansia muciniphila*

*A. muciniphila* ATCC BAA-835 genome was revealed firstly in 2011 with 2, 176 protein-coding sequences (van Passel et al., 2011). Low identity (14.6–28.8%) between genomes of representatives of the *Verrucomicrobia* phylum was indicated. Through secretome analysis, 61 proteins are annotated as glycosyl hydrolases, proteases, sulfatases, and sialidases, which are candidates involved in the degradation of mucin. Using enterobacterial repetitive intergenic consensus (ERIC-PCR) DNA fingerprinting method, 12 distinct clusters were distinguished among 22 strains identified as *A. muciniphila* from southern China healthy human (Guo et al., 2016). *A. muciniphila* strains isolated from different people may belong to different subtypes and the strains with two different subtypes were isolated from the feces of a single subject. Further studies are needed to explain the relationship between subtypes of *A. muciniphila* and human health. Comparative genomic analysis also revealed that 23 *Akkermansia* strains could form four clades in phylogenetic trees (Xing et al., 2019).

In 2017, Guo et al. characterized the genomic architecture of *A. muciniphila* using whole-genome sequencing and the analysis of 39 human and mouse feces isolates and reconstructed 106 draft genomes from available metagenomic datasets, and they identified three phylogroups through phylogenetic analysis (Guo et al., 2017). Three species-level phylogroups (AmI, AmII, and AmIII) had distinct metabolic and functional features. AmI was the most frequent phylogroup, which were found in 93% of human samples, 91% of mice and 9% of pigs. AmII is also commonly found in the human gut, with a higher incidence in Europeans (44%) than Chinese (27%) and Americans (33%). In 2020, Kirmiz et al. reported four species-level phylogroups (AmI to AmIV) with distinct functions through comparative genomic analysis (Kirmiz et al., 2020). Genes for cobalamin (vitamin B12) biosynthesis were identified within the AmII and AmIII phylogroups, and vitamin B12 production by the AmII phylogroup were confirmed. Vitamin B_12_ is a crucial component in host–microbe interactions due to limited availability and its importance in the human gut (Degnan et al., 2014). Hence, the difference in vitamin B_12_ production for different phylogroup strains may indicate different interactions.

Population genomics analysis showed varied geographical and species distribution for different subspecies (Amuc1 to Amuc4) of *A. muciniphila*. A large-scale population genomics analysis was conducted for the *Akkermansia* genus including 188 sequenced genomes of the isolates and 2,226 genomes assembled from metagenomes of humans and other animals (Karcher et al., 2021). The results indicated that *A. muciniphila* showed whole-genome divergence and was stratified in four subspecies from Amuc1 to Amuc4, among which Amuc1 is most prevalent in humans (47%), followed by Amuc2 and Amuc3 (27 and 24%). Human specific Amuc2 and Amuc3 are not found in mice and non-human primates, whereas Amuc1 and Amuc4 are present in both humans and mice. The prevalence of Amuc4 was more commonly found in non-Westernized human populations compared to non-Amuc4 species. Analysis of metagenome-assembled genomes of *Akkermansia* revealed that Amuc III mainly distributed in the Chinese population and Amuc IV was more commonly present in Western populations, whereas Amuc I and II distributed extensively globally (Lv et al., 2022). The representative genomes of Amuc I, II, III, and IV showed diversified genomic characteristics involved in multiple metabolism and transport pathways, which suggests different evolution history and functional habits. Becken et al. proposed that AmI can be divided into two related subclades (Ia and Ib) (Becken et al., 2021). The doubling times of AmI strains was faster, while that of AmII and AmIV strains was slower. Strains also showed differences in their sensitivity to ambient oxygen, AmII was oxygen resistant and AmIV was very sensitive to oxygen. Different oxygen sensitivities were also observed in AmIa and AmIb groups. AmIb strains were highly sensitive to air exposure, whereas AmIa strains were moderate resistance to air exposure (Becken et al., 2021). The AmIV strain had high adhesion ability to epithelial cells and showed a greater tendency to aggregate when growing in mucin medium. Phylogroups AmIV and AmII outcompeted AmI strains in antibiotic-treated mice. Hence, the genetic and phenotypic diversity of *A. muciniphila* strains may be an important variable to consider when inferring the influences of this microbe on host health.

## 4 Antibiotic resistance characteristics of *Akkermansia muciniphila*

Antibiotic resistance of *A. muciniphila* is an important safety concern in its clinical application for disease treatments. Guo et al. compared the genomes of 40 *A. muciniphila* strains (39 newly isolates and ATCC BAA-835) with other genomes from the NCBI database, and reported the lateral gene transfer of eight genes between *A. muciniphila* GP36 and *Salmonella enterica* including three antibiotic resistance genes (Guo et al., 2017). These genes are *sul2* gene encoding sulfonamide-resistant dihydropteroate synthase, *aph(6)-Id* and *aph(3″)-Ib* gene encoding aminoglycoside phosphotransferase. Drug sensitive test was analyzed for *A. muciniphila* GP36 and ATCC BAA-835 including amikacin, sulfonamides, teicoplanin, polymyxin, cefoperazone-sulbactam, meropenem and minocycline. ATCC BAA-835 was resistant to teicoplanin and sensitive to other antibiotics, whereas *A. muciniphila* GP36 was resistant to amikacin, sulfonamides and teicoplanin. These results indicated the *A. muciniphila* might acquire antibiotic resistance via lateral gene transfer. Dubourg et al. reported that *A. muciniphila* Muc^T^ strain was susceptible to imipenem, piperacillin/tazobactam and doxycycline, whereas was resistant to metronidazole (MIC >64 mg/L), vancomycin (MIC >64 mg/L) and penicillin G (MIC = 2.8 mg/L) (Dubourg et al., 2013). Antimicrobial susceptibility analysis indicated the resistance of *A. muciniphila* DSM 22959 (ATCC BAA-835) to chloramphenicol, clindamycin, streptomycin and erythromycin, whereas the strain was sensitive to ampicillin, tetracycline, gentamicin and kanamycin (Cozzolino et al., 2020). Machado et al. reported that *A. muciniphila* DSM 22959 strain was resistant to gentamicin, kanamycin, streptomycin (aminoglycosides) and ciprofloxacin (fluoroquinolones), whereas was susceptible to ampicillin, tetracycline, colistin, and fosfomycin (Machado et al., 2022). Opposite susceptibility results of gentamicin and kanamycin were reported for *A. muciniphila* DSM 22959 possibly due to different cut-off values and growth media used in the two studies.

Whether there is a risk of horizontal transfer of drug resistance genes is also an issue that needs to be considered in probiotic development. Machado et al. analyzed the genomes of 189 *A. muciniphila* strains and reported the existence of antibiotic resistance genes (ARGs) related with resistance to macrolides, fosfomycin, aminoglycosides, tetracyclines, and β-lactams (Machado et al., 2022). The ARGs of *A. muciniphila* DSM 22959 is consistent with the phenotypic feature for partial antibiotic resistance, while no resistant phenotypes were observed for the genes related with β-lactams, tetracyclines and fosfomycin resistance. Meanwhile, analysis of the genome sequences indicated that *A. muciniphila* DSM 22959 posed little risk of ARG horizontal transfer because there is no mobile genetic elements detected within its genome. Similar results were reported for the type-strain Muc^T^ and human isolates of *A. muciniphila*, intrinsic resistance genes observed seem to pose no risks by determining their antibiotic resistance phenotype, and there is no significant risk for the horizontal transfer of ARGs (Ouwerkerk et al., 2022). These results indicated the ARGs of *A. muciniphila* might pose a small risk of transmission.

Filardi et al. evaluated the antibiotic susceptibility of five human isolated *A. muciniphila* strains and found that one strain harboring *tetW* gene showing tetracycline resistance (Filardi et al., 2022). All five *A. muciniphila* strains had low sensitivity to ciprofloxacin and aminoglycosides including gentamicin, kanamycin and streptomycin, whereas no related antibiotic resistance genes were found in the genome. The gene *adeF* encoding one component of the resistance-nodulation-cell division efflux pump AdeFGH system was detected on the genomes of the isolates, whereas the treatment using efflux pump inhibitors did not alter the antibiotic susceptibility of the strains to ciprofloxacin. Hou et al. evaluated the safety of healthy human *A. muciniphila* isolates (AM01 to AM31) from feces and breast milk (AM06) as a probiotic (Hou et al., 2023). About 13 or 14 ARGs were predicted for AM01 to AM06 using Antibiotic Resistance Genes Database (ARDB), whereas Resistance Gene Identifier (RGI) analysis indicated only one antibiotic resistance gene in the genomes of AM01, AM04, AM05 and AM06, and two antibiotic resistance genes in the genomes of AM02 and AM03. The antibiotic susceptibility analysis indicated that all the strains were resistant to vancomycin, gentamicin, teicoplanin, ofloxacin, norfloxacin and bacitracin, whereas not all strains were resistant to cefoperazone, penicillin, and chloramphenicol, although they harbor related resistance genes. AM02 and AM03 showed resistance to lincosamide. In addition, AM01 and AM04 to AM06 showed resistance to kanamycin and ciprofloxacin, although no related antibiotic resistance genes were predicted. Hence, the presence of ARGs in the bacterial genome does not necessarily result in a resistant phenotype; more phenotype studies are required to confirm the antibiotic susceptibility of the strain especially for the new isolates.

In summary, current findings highlight the urgent need for standardized breakpoints and protocols to assess the antimicrobial sensitivity of *A. muciniphila* strains and to ensure comparability of results across different studies. Meanwhile, future studies should focus on clarifying the transferability risk of resistance genes. In addition, further studies on additional strains other than type strain of *A. muciniphila* are imperative to confirm the safety of this microbe in further application.

## 5 *Akkermansia muciniphila* and infection related diseases

### 5.1 *Akkermansia muciniphila* and sepsis

**Figure 2:**
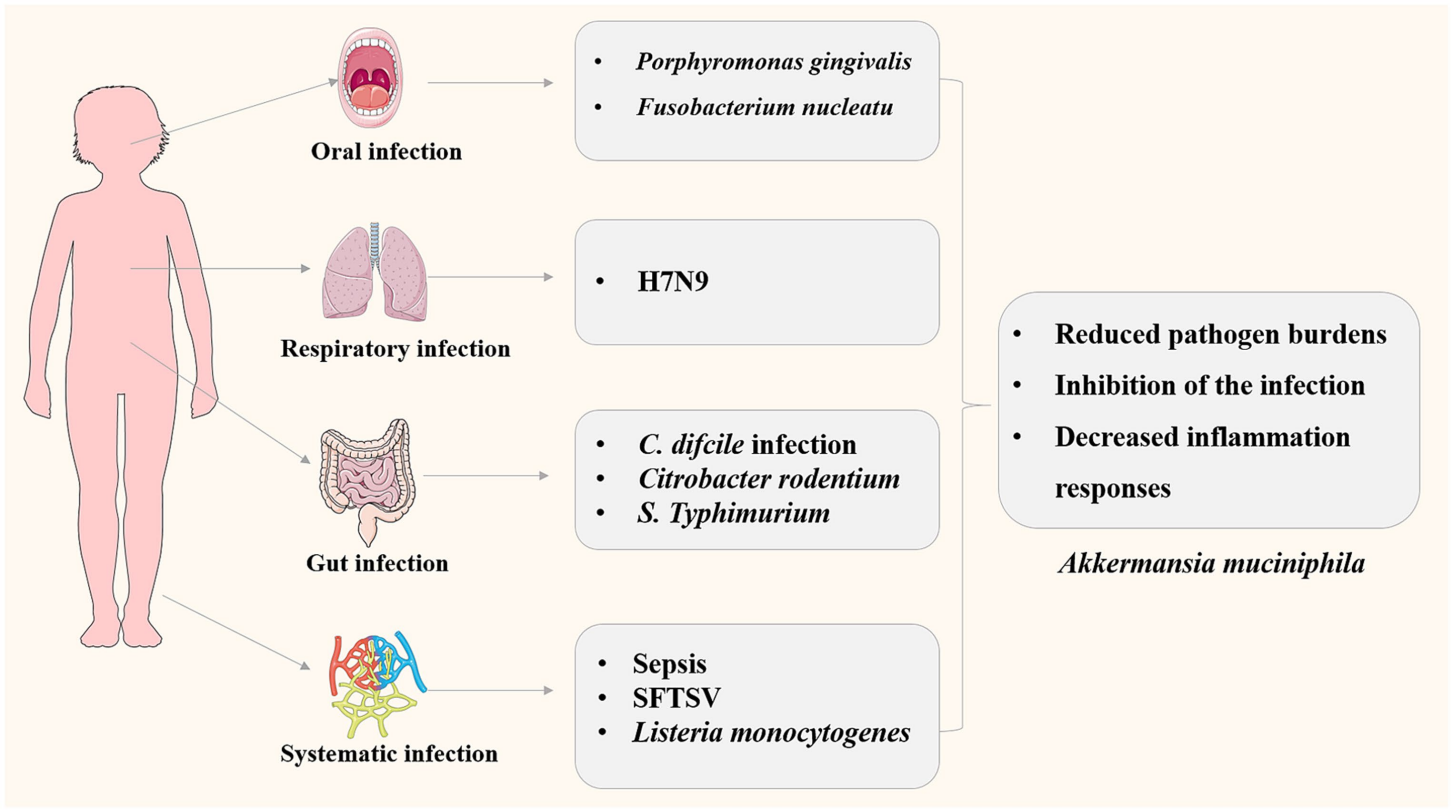
Roles of *A. muciniphila* in infectious diseases involving different pathogens and different systems.

Sepsis is a life-threatening organ dysfunction caused by an unbalanced host response to infection and is a major medical burden worldwide (van der Poll et al., 2017). The pathogenesis of sepsis is closely related to intestinal flora, and clinical treatments for sepsis are still limited. Statistically, nearly 20% of deaths reported globally are due to sepsis (Rudd et al., 2020). *A. muciniphila* derived tripeptide RKH protects against lethal sepsis was reported recently and the mechanism was revealed (Xie et al., 2023) (Table 1; Figure 2). Compared with non-septic controls, gut *A. muciniphila* abundance in septic murine model significantly reduced. Interestingly, supplementation of both live *A. muciniphila* and its culture supernatant could significantly reduce the mortality of sepsis models. Live *A. muciniphila* and its supernatant could protect against sepsis associated organ damage and reduce pulmonary inflammation, whereas heat-killed *A. muciniphila* could not. Metabolomics analysis indicated elevated expression of a novel tripeptide Arg-Lys-His (RKH) in live *A. muciniphila* supernatant. Meanwhile, fecal RKH levels were significantly lower in patients with sepsis compared to healthy controls. RKH pretreatment could significantly extend the survival time of septic mice by alleviating acute tissue injuries and reducing inflammatory factor expression. RKH treatment suppressed the expression of proinflammatory cytokines in macrophages including bone marrow-derived macrophages (BMDMs) and human monocyte-derived macrophages (THP-1-dMs) after LPS stimulation. RKH inhibits systemic inflammation during sepsis through directly binding to the Toll-like receptor 4. Protection of RKH was also verified using a septic piglet model, and safety assessment indicated no obvious adverse effects *in vivo*. Hence, a novel tripeptide RKH produced by live *A. muciniphila*, may serve as a new promising treatment approach to combat lethal sepsis, which may need further evaluation before transformation into clinical practice.

**Table 1:** Reports on the function and action mechanisms of *A. muciniphila* on infectious diseases.

Diseases	Disease models	Forms of *A. muciniphila*	Effects	Action mechanism	References
Sepsis	Septic murine model; a septic piglet model	Live *A. muciniphila*, culture supernatant; tripeptide RKH	Alleviate acute tissue injuries and reduce sepsis-induced mortality	Reducing inflammatory factor expression; RKH inhibits systemic inflammation through directly binding to the Toll-like receptor 4 receptor.	Xie et al. (2023)
Influenza	H7N9 infection mouse model	Cultures and pasteurized *A. muciniphila*	Reduced weight loss and mortality	Reducing IL-1β and IL-6 levels, enhancing IFN-β, IFN-γ, and IL-10 expression in H7N9-infected mice.	Hu et al. (2021)
COVID-19	COVID-19 patients	Live *A. muciniphila*	*A. muciniphila* abundance elevated in the COVID-19 patients	*A. muciniphila* abundance positively correlated with inflammatory cytokines IL-1β and IL-6 and CXCL8.	Yeoh et al. (2021)
Severe fever with thrombocytopenia syndrome	SFTSV infection patients; Abx mice infection model	Live and pasteurized *A. muciniphila*	Akk abundance reduced in samples from deceased SFTS patients	Reduced serum expression of IL-1β, IL-6 and TNF-α; HAL regulates primary BA conjugation and protects host against SFTSV infection by suppressing NF-κB-mediated systemic inflammation.	Xie et al. (2023)
*C. difcile* infection	Mice infection model	Live *A. muciniphila*	Improve clinical outcomes of CDI mice	Increased intestinal barrier by increasing the expression of tight junction proteins; Reduced local and systemic immune response (reduced expression of IL-6, TNF-α, IL-1β, CCL1, CCL2, CCL3, CCL4, CCL5, CCL17, CCL22, CXCL10, and CXCL13); Altered autophagy and innate immunity in the colon, alleviated microbiome dysbiosis, and improved bile acid and SCFA metabolism.	Wu et al. (2022)
*C. difcile* infection	Caco-2 cells	Live, UV-killed, cell-free supernatant and extracellular vesicles	Inhibition of cytotoxicity and inflammatory response	Reduced the expression of IL-1β, TNF-α, and IL-10 in Caco-2 cell model; Changed the expression of gut barrier–related genes and inflammatory response.	Nasiri et al. (2023)
*Citrobacter rodentium* induced colitis	Mice infection model	Live *A. muciniphila*	Reduce the symptoms and pathological changes	Enhanced mucus barrier (upregulated expressions of gene encoding mucin, including muc1, muc5, and muc13) and anti-microbial responses (upregulation of Reg3γ, CRAMP and IL-22).	Mao et al. (2021)
Periodontitis (*Porphyromonas gingivalis*)	Mice infection model	Live, pasteurized *A. muciniphila;* Amuc_1100	Decreased periodontal destruction and systemic inflammation	Increased anti-inflammatory effects (increased IL-10 and decreased IL-12); Improved expression of tight junction molecules (ZO-1) and cell–cell adhesion markers; Increased anti-infective response by upregulating IL-8 expression.	Mulhall et al. (2022) and Huck et al. (2020)
Periodontitis (*Fusobacterium nucleatum*)	Mice infection model	Live *A. muciniphila*	Inhibition of the periodontitis	Inhibit TLR/MyD88/NF-κB pathway and secretion of inflammatory factors.	Song et al. (2023)
*S. typhimurium* infection	Gnotobiotic C3H mouse model	Live *A. muciniphila*	Exacerbating infectious and inflammatory symptoms	Increased expression levels of IFN-γ, IP-10, TNF-α, IL-12, IL-17 and IL-6 in the cecal and colonic tissue of the mice.	Ganesh et al. (2013)
*S. typhimurium* infection	Streptomycin-treated C57B6J mouse infection model	Live and pasteurized *A. muciniphila*	Reduced fecal and systemic pathogen burdens and decreased inflammation responses	AKK promotes the expression of intestinal barrier genes and the secretion of antimicrobial peptides. pAkk promotes NLRP3 expression, and enhances the antimicrobial activity of macrophage through increased production of NO, ROS, and inflammatory cytokines.	Liu et al. (2023)
*Listeria monocytogenes* infection	High-fat/westernized diet mouse infection model	Live *A. muciniphila*	Reduce systemic infection	*A. muciniphila* ameliorated inflammatory gene expression (decreased expression of TNFα and Foxp3, and elevated expression of Ccl2) in the distal ileum.	Keane et al. (2023)

### 5.2 *Akkermansia muciniphila* and virus infection

Influenza is a global infectious disease caused by a single stranded negative sense RNA virus named influenza virus, which is Liu et al. (2023). According to the statistical data of World Health Organization, seasonal influenza epidemics cause an estimated 3 to 5 million cases of severe cases worldwide, resulting in approximately 500,000 deaths, and causing significant economic losses and social burdens (Eichberg et al., 2022). H7N9 influenza virus, an emerging zoonotic pathogen, has led to 1, 564 laboratory-confirmed cases of human infection from its initial outbreak until October 2017, and there are still sporadic infections now (Liu et al., 2023). Hu et al. reported that *A. muciniphila* improved host defense against influenza virus H7N9 infection (Hu et al., 2021). H7N9 infection could affect mouse gut microbiota including the increase of *A. muciniphila* abundance. Oral administration of pasteurized *A. muciniphila* (1 × 10^8^ CFU) and its cultures significantly reduced weight loss and mortality of H7N9 infected mice, by reducing pulmonary viral titers, decreasing IL-1β and IL-6 levels, enhancing IFN-β, IFN-γ, and IL-10 expression. Hence, *A. muciniphila* could enhance host anti-influenza role through improving the innate immune response to H7N9 infection by regulating anti-inflammatory and immunoregulatory properties (Table 1).

COVID-19, caused by severe acute respiratory syndrome-Coronavirus 2 (SARS-CoV-2), is a pandemic that has affected the globe, leading to nationwide lockdowns. Research suggests that the gut microbiome may be a key factor in regulating host response and disease severity in COVID-19 patients (Aggarwal et al., 2022). Yeoh et al. analyzed the gut microbiota composition and host immune response markers in patients with COVID-19, and found that *A. muciniphila* abundance elevated in the COVID-19 patients (Yeoh et al., 2021). Moreover, the abundance of *A. muciniphila* positively correlated with inflammatory cytokines IL-1β and IL-6 and proinflammatory cytokine C-X-C motif ligand 8 (CXCL8). The cytokine storm was reported to be related with COVID-19 severity (Han et al., 2020). Hence, *A. muciniphila* may participate in the pathogenesis of COVID-19 and more researches are required.

Severe fever with thrombocytopenia syndrome (SFTS) is an tick-born infectious disease caused by a negative-strand RNA virus belonging to *phlebovirus*, which is originally reported in mainland China in 2009 (Yu et al., 2011). About 13, 305 patients have been diagnosed with SFTS in China until December 2020 (Che et al., 2022). Symptoms of SFTS include fever, thrombocytopenia, and leukopenia, with a fatality rate ranged from10 to 30% (Zhuang et al., 2018). Xie et al. reported an *A. muciniphila*–BA–TGR5 axis that regulates host NF-κB-mediated immunopathogenic responses to SFTSV infections (Xie et al., 2023). Relative abundance of *A. muciniphila* increased during the course of SFTSV infection in the surviving patients compared with heathy controls, whereas *A. muciniphila* abundance reduced in samples from deceased SFTS patients (SF-D group) compared with surviving SFTS patients (SF-S group). SF-S patients had reduced serum levels of the proinflammatory cytokines IL-1β, IL-6 and TNF-α compared with SF-D patients. The proinflammatory cytokine levels was inversely related with the relative *A. muciniphila* abundance. Intragastric administration of live and pasteurized *A. muciniphila* showed significant protection for Abx mice (microbiota-depleted mice) against fatal SFTSV infection compared with controls of unrelated commensal bacteria. The β-carboline alkaloid harmaline (HAL) generated by *A. muciniphila* can regulate primary BA conjugation and protect host against SFTSV infection through the inhibition of NF-κB-mediated systemic inflammation.

### 5.3 *Akkermansia muciniphila* and enteric infectious diseases

*Clostridioides difcile* (*C. difcile*) is a Gram-positive anaerobic bacterium, which can cause *C. difcile* infection (CDI) in healthcare facilities, with a high CDI recurrence rate of 15–35% (Finn et al., 2021). Gut microbe diversity is associated with the recurrence and severity of CDI (Maziade et al., 2015). Probiotics are widely recommended for the prevention of CDI and its recurrence, and the protective roles and underlying mechanisms of *A. muciniphila* on CDI were reported (Wu et al., 2022; Nasiri et al., 2023). Wu et al. reported that oral supplementation of *A. muciniphila* could reduce *C. difficile* burden and its toxins, and improve clinical outcomes of CDI mice including reduced body weight loss, alleviated diarrhea, relieved colon shortening (Wu et al., 2022). The protection mechanisms of *A. muciniphila* on CDI include increasing intestinal barrier by increased tight junction protein expression, reduced local and systemic immune response (reduced expression of IL-6, IL-1β, TNF-α, CCL1, CCL2, CCL4, CCL5, CCL3, CXCL10, CCL17, CCL22, and CXCL13), changing autophagy and innate immunity, alleviating microbiome dysbiosis, and improving bile acid and short-chain fatty acids (SCFAs) metabolism. The expression of autophagy related proteins (light chain 3 (LC3)-II, beclin1, autophagy-related gene 5 (Atg5), Atg9a, Atg7, and Atg12) and immune markers (, cluster of differentiation 14 (CD14), TLR4 and myeloid differentiation 88, MyD88) downregulated in the *A. muciniphila* group. Nasiri et al. reported that *A. muciniphila* and its derivatives could suppress cytotoxicity and inflammatory response induced by *C. difcile* RT001 *in vitro* using Caco-2 cells (Nasiri et al., 2023). Compared with untreated controls, the survival rate of Caco-2 cells treated with live, UV-killed, cell-free supernatant (CFS, 10^6^ cfu/mL), and extracellular vesicles (EVs, 20 μg/mL) of *A. muciniphila* exceeded 90%. The treatment exerted function by reducing the expression of IL-1β, TNF-α, and IL-10 in Caco-2 cell model, and changing the expression of gut barrier–related genes and inflammatory response. Therefore, EVs and CFS of *A. muciniphila* may be a safe substitution to live bacteria that can prevent harmful effects of *C. difficile* toxins, which merits further *in vivo* verifications.

Protective roles of *A. muciniphila* were reported for another intestinal pathogen *Citrobacter rodentium*, which can induce bacterial colitis (Mao et al., 2021). Increased *A. muciniphila* abundance was correlated with the alleviation of *C. rodentium* infection and intestinal inflammation in the hyaluronan treated mice. *A. muciniphila* is the key species responding to hyaluronan treatment, and fecal transplantation experiments demonstrated the transferable of hyaluronan induced microbiome. *A. muciniphila* colonization in mice can significantly reduce the symptoms and pathological changes of *C. rodentium* infection, with less body weight loss, pathogen tissue loads and reduced proinflammatory cytokine (IL-1β) expression. The protective function of *A. muciniphila* on *C. rodentium* induced-colitis is implemented throughenhanced mucus barrier (upregulated mucin gene expression including *muc1*, *muc5*, and *muc13*) and anti-microbial responses (upregulation of Reg3γ, CRAMP, and IL-22). Together, these results indicate that *A. muciniphila* acts as regulator of gut barrier and immune responses to enteric infectious diseases.

### 5.4 *Akkermansia muciniphila* and periodontitis

Periodontitis, which occurs in the periodontal support tissue, is a chronic inflammatory disease that could lead to tooth loss in adults, and severe periodontitis has become the sixth most prevalent disease worldwide (Slots, 2017; Tonetti et al., 2017). *Porphyromonas gingivalis* is a gram-negative anaerobe and a main pathogen of periodontitis (Shalihin et al., 2023). It was demonstrated that oral gavage with pasteurized *A. muciniphila* decreased *P. gingivalis*-associated periodontal destruction and ameliorated systemic inflammation in lean and obese mice (Huck et al., 2020; Mulhall et al., 2022). Oral administration of *A. muciniphila* and its pili-like protein Amuc_1100 could reduce alveolar bone loss and inflammatory destruction in murine periodontitis models (Huck et al., 2020). *A. muciniphila* and *P. gingivalis* co-culture resulted in decreased expression levels of *P. gingivalis* virulence factor gingipains and increased expression of *A. muciniphila* Amuc_1100. The protection of *A. muciniphila* may act through increasing anti-inflammatory effects (increased IL-10 and decreased IL-12) in Mouse bone marrow macrophages (BMMϕ), improving the expression level of tight junction molecules (ZO-1) and adhesion markers, and increasing anti-infective response by upregulating IL-8 expression in gingival epithelial cells. Interestingly, a similar protective effect was found for oral administration of pasteurized *A. muciniphila*, viable *A. muciniphila* and Amuc_1100 (Mulhall et al., 2022). The route of administration is key to preventing tissue destruction, as gavage does not significantly reduce periodontal destruction. The use of pasteurization *A. muciniphila* has more potential in an industrial point of view because it has minimal safety concerns and the same beneficial effects as live *A. muciniphila*.

Later, Song et al. reported an inhibition of *A. muciniphila* on the periodontitis caused by *Fusobacterium nucleatum* (Song et al., 2023). It has been verified that *F. nucleatum* can cause periodontitis and copolymerization with other periodontal pathogens, making it an important target for the prevention of periodontitis (Park et al., 2016). Bacterial co-culture experiments showed that *A. muciniphila* could restrain virulence gene expression of *F. nucleatum* by inhibiting TLR/MyD88/NF-κB pathway and inflammatory factor secretion. Finally, inhibition of *A. muciniphila* on the periodontitis caused by *F. nucleatum* was verified using BALB/c mice experiments. Therefore, *A. muciniphila* may act as a potential therapeutic strategy for periodontitis, which could inhibit the virulence factors of the pathogen causing periodontitis and reduce the immune response of the host.

### 5.5 *Akkermansia muciniphila* and foodborne infection

Nontyphoidal *Salmonella enterica subsp. enterica serovars* (*S. typhimurium*) is an intracellular bacterial pathogens causing hundreds of thousands of acute gastroenteritis cases each year (Vieira et al., 2022). There are two opposing reports about the effect of *A. muciniphila* on *S. typhimurium* infection in mouse model. Ganesh et al. reported that *A. muciniphila* exacerbated gut inflammation of *S. typhimurium* infection (Ganesh et al., 2013). A well-defined gnotobiotic C3H mouse model with a defined simplified human intestinal microbiota (SIHUMI) of eight bacterial species was used to analyze the influence of *A. muciniphila* on inflammatory and infectious symptoms caused by *S. typhimurium.* Additional *A. muciniphila* colonization in *S. typhimurium*-infected C3H mouse model significantly elevated histopathology scores and increased expression levels of IFN-γ, TNF-α, IL-12, IP-10, IL-17, and IL-6 in the cecal and colonic tissue of the mice, thereby exacerbating infectious and inflammatory symptoms. However, Liu et al. reported that *A. muciniphila* could decrease mice susceptibility to *S. typhimurium* infection (Liu et al., 2023). A streptomycin-treated C57B6J mouse infection model was constructed to evaluate the impact of live *A. muciniphila* (AKK) and pasteurized *A. muciniphila* (pAKK) on *S. typhimurium* infection. The results indicated that AKK and pAKK pretreatment significantly reduced pathogen burdens and decreased inflammation responses during *S. typhimurium* infection. There may have different protective mechanisms for AKK and pAKK treatments. Analysis of SCFAs levels indicated higher propionate levels in AKK-treated group compared to control or pAKK-treated mice. Live *A. muciniphila* promotes the expression of intestinal barrier genes and antimicrobial peptide secretion, and co-housing studies have shown that *A. muciniphila*-associated microbial communities play a role in alleviating infection symptom. The researchers verified that pAKK pretreatment could promote NLRP3 expression, and increase the antimicrobial activity of macrophage, possibly through increased production of nitric oxide (NO), reactive oxygen (ROS) and inflammatory cytokines. Differences between the composition of microbial community and infection conditions in mice could explain the opposite phenotypes observed between two studies, and further research is required to reveal the roles and mechanisms of *A. muciniphila* in *S. typhimurium* infection.

*Listeria monocytogenes* is a Gram-positive foodborne pathogen that causes mild to severe gastroenteritis in healthy individuals, whereas can cause bacterial sepsis, bacterial meningitis in children, elderly individuals, and immunocompromised individuals (Radoshevich and Cossart, 2018). *L. monocytogenes* is rod-shaped facultative anaerobes, which can tolerate low temperatures and has high resistance to environmental stresses, making it a major concern for the food industry (Gandhi and Chikindas, 2007). The effects of *A. muciniphila* on *L. monocytogenes* infection in the high-fat/westernized diet mice were investigated (Keane et al., 2023). *A. muciniphila* treatment could reduce systemic *Listeria* infection induced by diet by reducing the bacterial loads in the liver, spleen and mesenteric lymph nodes, demonstrating that oral gavage with *A. muciniphila* enhances mice *L. monocytogenes* resistance. The molecular mechanism may be that *A. muciniphila* ameliorated inflammatory gene expression (decreased expression of Tnfα and Foxp3, and elevated expression of Ccl2) in the distal ileum thus leading to a reduction in inflammatory cell infiltration. The results indicated potentials for the use of microbial interventions in the prevention of foodborne infectious diseases. The roles of *A. muciniphila* in infection related diseases were summarized in Figure 2.

## 6 Interaction mechanism between *Akkermansia muciniphila* and host

### 6.1 Regulation of host immune response and inflammation

**Figure 3:**
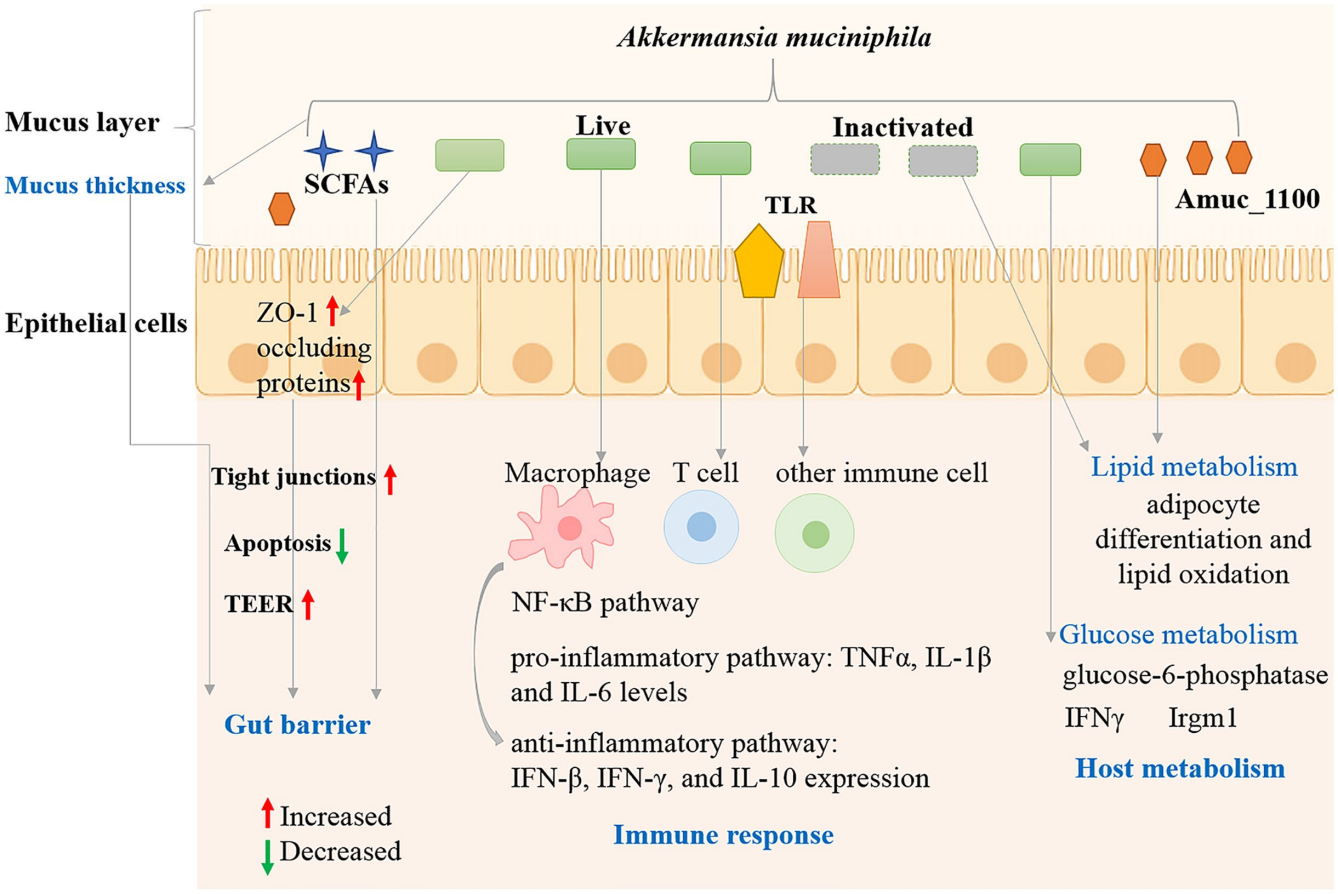
Interaction mechanisms between *A. muciniphila* and host.

Intestinal immune barrier refers to innate and adaptive immune cells and gut-associated lymphoid tissue colonized on the intestinal lamina propria (Di Tommaso et al., 2021). Environmental factors, intestinal flora and their metabolites could be recognized by specific receptors (toll-like receptors, TLRs) on immune cells, leading to intestinal immune homeostasis or imbalance (Shi et al., 2023). Multiple investigations have shown that *A. muciniphila* plays an important role in regulating the immune response and host inflammation. The regulatory roles could complete by a variety of forms such as live or inactivated bacteria, culture supernatant or derived components of *A. muciniphila.* Germ-free mice were utilized to analyze the influence of *A. muciniphila* on the host response (Derrien et al., 2011). *A. muciniphila* colonized most in the cecum, which produce the most mucin. Global transcriptional analysis indicated that *A. muciniphila* changed mucosal gene expression profiles by increasing the expression of genes involved in immune responses and cell fate. Exposure to SCFAs generated by *A. muciniphila* could alter the gene transcriptional levels in mouse ileal organoid model (Lukovac et al., 2014). The results indicated that *A. muciniphila* and its metabolites had an impact on the expression of transcription factors and genes involved in cellular lipid metabolism and growth. The β-carboline alkaloid harmaline (HAL) generated by *A. muciniphila* can regulate primary BA conjugation and suppressed NF-κB-mediated systemic inflammation (Xie et al., 2023). Oral administration of cultures and pasteurized *A. muciniphila* (1 × 10^8^ CFU) significantly reduced IL-6 and IL-1β levels, enhanced IFN-γ, IFN-β, and IL-10 expression in H7N9-infected mice (Hu et al., 2021). The inhibitory effects of *A. muciniphila* and its derivatives on cytotoxicity and inflammatory response were also reported for *C. difcile* RT001 induced Caco-2 cells (Nasiri et al., 2023). In summary, protective effect *of A. muciniphila* and its derivatives is inversely associated with inflammatory status and aids the immune response by regulating anti-inflammatory pathway (Figure 3).

Adaptive immune cells play an important role in the protection of intestinal mucosal barrier and tissue homeostasis through immunoglobulin A (IgA) (Belkaid and Harrison, 2017). Mouse studies have shown that *A. muciniphila* can specifically induce immune responses to T cells during homeostasis (Ansaldo et al., 2019). *A. muciniphila* has been shown to induce IgG1 and T cell-related immune responses in mice, T-cell response induced by *A. muciniphila* could also occur independently through follicular T-cell. Amuc_1100 protein is a pili-like protein highly abundant in the outer membrane of *A. muciniphila* Muc^T^, which could activate the NF-κB pathway through activation of receptors such as Toll-like receptor (TLR) 2 and TLR4 (Ottman et al., 2017). Recently, a phospholipidlipid was identified from the cell membrane *A. muciniphila*, with immunomodulatory activity in cell-based assays through toll-like receptor TLR2–TLR1 heterodimer (Bae et al., 2022). The phospholipidlipid could induce pro-inflammatory cytokines IL-6 and TNFα expression. Moreover, it can reset the activation threshold of dendritic cells and regulate the immune stimulation. Dendritic cells typically recognize and respond to bacterial metabolites through the pathogen-associated molecular pattern (PAMP) receptors such as TLR2 and TLR4 (Kang et al., 2009).

Besides T cells, *A. muciniphila* can also influence the intestinal immune through regulating the function of other immune cells (Figure 3). *A. muciniphila* derived tripeptide RKH could suppress the expression of proinflammatory cytokines in macrophages after LPS stimulation and directly bind to TLR4 and inhibit systemic inflammation of sepsis (Xie et al., 2023). Treatment of peripheral blood mononuclear cells (PBMCs) with *A. muciniphila* (live cells, heat-killed cells and supernatant) induce production of both anti-inflammatory and pro-inflammatory cytokines (IL-6, IL-1β, IL-8, IL-10, and TNF-α) (Ottman et al., 2017).

### 6.2 Enhancement of the intestinal barrier function

The intestinal barrier is a complex and well-organized physiological structure, which interacts with the external environment as a biochemical, physical, and immune barrier (Breugelmans et al., 2022). In healthy conditions, the intestinal barrier is semi-permeable, allowing the absorption of nutrients and water and protecting the internal environment from potential penetration by pathological molecules and microorganisms (Maynard et al., 2012). However, the damage of the intestinal barrier integrity results in multiple local and systemic diseases. Intestinal mucus consists of an inner layer without bacteria and a thicker outer layer with symbiotic bacteria (Hansson and Johansson, 2010). The integrity of the intestinal barrier requires normal epithelial boundary, maintenance of tight junctions, normal mucus secretion and a normal gut microbiome, as well as a finely regulated immune system (Shi et al., 2023).

Mucins, composed of amino acids and oligosaccharides, is a nutrient source for intestinal bacteria anda gatekeeper of the gastrointestinal mucosal barrier (Breugelmans et al., 2022). *A. muciniphila* bacteria can grow using mucins as sole carbon and nitrogen source in the intestinal mucus layer, and settle in the intestinal tract through competitive rejection and protect the intestinal tract from pathogens (Figure 3). The presence of mucin could increase the expression levels of mucin-degrading enzymes, whereas most genes involved in glycolysis and energy metabolic pathways upregulated under low mucin conditions (Shin et al., 2019). *A. muciniphila* abundance was positively related with mucin content in the cecum (van den Abbeele et al., 2011). Antibiotic treatment increased the mucus barrier by reducing the abundance of mucin-degrading *A. muciniphila* and decreasing Muc2 gene (encoding the major mucin of the colonic mucus in colonic tissues) expression (Ijssennagger et al., 2015). Mucin levels in the small intestine increased indicated by increased expression of MUC5 and MUC2 after metformin treatment in female mice, and thickened intestinal mucosa was confirmed by immunohistochemical assays and *A. muciniphila* abundance increased (Lee and Ko, 2014). *A. muciniphila* abundance was positively associated with the number of mucin-producing goblet cells in mice after metformin treatment (Shin et al., 2014). *A. muciniphila* also simulates mucin production in addition to its ability to degrade mucin. Therefore, although *A. muciniphila* make use of mucin as an energy source, it can positively regulate the thickness and stability of intestinal mucus layer and the intestinal barrier integrity.

In addition to affecting the mucin layer, other mechanisms may affect the integrity of the intestinal barrier (Figure 3). The interaction between *A. muciniphila* and the host might influence immune tolerance and homeostasis in the gut (Everard et al., 2013). Viable *A. muciniphila* administration elevated the levels of intestinal endocannabinoids and improved the metabolic profile and the mucus layer thickness (Everard et al., 2013). Endocannabinoids play roles in the gut inflammation, gut peptide secretion and the gut barrier. *A. muciniphila* and Amuc_1100 increased trans-epithelial resistance (TEER) in Caco2-cells, indicating their roles in strengthening the epithelial barrier function (Ottman et al., 2017).

Tight junctions are the connections between intestinal epithelial cells, which consist of zonula occluden (ZO), claudins (Cldns) and occludin (Ocln) proteins, are critical for normal function of epithelial cells and maintenance of intestinal barrier functions (Allam-Ndoul et al., 2020). *A muciniphila* could increase the expression of intestinal tight junction proteins including ZO-1 and occluding proteins, thereby decreasing Western diet-induced gut permeability and contributing to the gut barrier function (Li et al., 2016). Luo et al. reported that active or autoclaved *A. muciniphila* could induce the expression of tight junction proteins (ZO-1 and occluding proteins) in intestinal epithelial cells (Luo et al., 2021). The apoptosis rate can reflect the degree of cellular damage, and *A. muciniphila* treatment significantly suppressed the apoptotic rate of the inflammatory IPEC-J2 cells, thus reducing the degree of cellular damage. Therefore, *A. muciniphila* may protect intestinal barrier integrity by regulation of epithelial cells, mucus secretion, tight junctions, normal gut microbiota and cellular damage level.

### 6.3 Metabolic regulation and nutrition of the intestinal wall

*A. muciniphila* genome encodes a large number of mucin-degrading enzymes, which can degrade mucin and generate short-chain fatty acids, such as butyrate, propionate, acetate, etc., playing important roles in the regulation of host metabolism and disease development (Cani and de Vos, 2017). Live, pasteurized bacterium and Amuc_1100 protein could improve metabolism in obese and diabetic mice (Plovier et al., 2017). Interestingly, pasteurized *A. muciniphila* showed stronger impacts on glucose intolerance, body weight and fat mass gain and in HFD-fed mice, which was associated with modulation of host urinary metabolomics and energy absorption of the intestinal tract. Everard et al. reported the roles of *A. muciniphila* in metabolic regulation including adipose tissue metabolism, fat storage and glucose metabolism in diet-induced obesity mouse model (Everard et al., 2013). *A. muciniphila* therapy completely reversed diet-induced fasting hyperglycemia via decreasing hepatic glucose-6-phosphatase expression, and affected adipose tissue metabolism by increasing the mRNA expression of adipocyte differentiation and lipid oxidation markers. Meanwhile, the colonization of *A. muciniphila* in IFNγKO and wild-type mice can improve host glucose metabolism was reported and verified in humans (Greer et al., 2016). IFNγ may control gut *A. muciniphila* levels by regulating Irgm1 gene expression in the mouse ileum. The relation between IFNγ, *A. muciniphila* and glucose tolerance exists in humans, suggesting a conserved mechanism in the regulation of metabolic health in mice and humans. Hence, *A. muciniphila* played roles in host metabolism regulation including glucose and lipid metabolism (Figure 3).

## 7 Issues to be considered in the application of *Akkermansia muciniphila*

The general characteristics of *A. muciniphila* strains from different origins, the efficacy of the strain on the diseases, the antibiotic resistance and the toxicity on the host should be evaluated completely before the application of *A. muciniphila*. Most reports on *A. muciniphila* treatment are conducted *in vitro* or using animal model, hence clinical trials must be conducted to confirm the safety and the efficacy of this promising NGP. Different studies may have reported opposite therapeutic effects, such as the effect of *A. muciniphila* on *S. typhimurium* infection (Ganesh et al., 2013; Liu et al., 2023), which is also worth further research to verify. The phenotype of antibiotic resistance to and the possible horizontal transfer of resistance genes also need more research. The resistance of different *A. muciniphila* strains to different antibiotics was different. Different susceptibility results of gentamicin and kanamycin were reported for *A. muciniphila* DSM 22959 due to the reference to different cut-off values and the methods used (Cozzolino et al., 2020; Machado et al., 2022). Hence, there is urgent need for standardized protocols and breakpoints to evaluate the antimicrobial sensitivity of *A. muciniphila* strains. More researches are required to access the antibiotic resistance of *A. muciniphila* strains isolated from different humans and different samples besides the type strain. One aspect is that as a gut microbe *A. muciniphila* starts to colonize in healthy subjects early in life and is also detected in breast milk and blood of human (Collado et al., 2007; Dubourg et al., 2017; Hou et al., 2023). Increased abundance of *A. muciniphila* may affect different systems in different individuals. The use of *A. muciniphila* derived bioactive molecule may be an alternative. In addition, the regulatory approval for NPG is also a concern in the drug development. In the US, the definition of live biotherapeutic products (LBP) presented by Food and Drug Administration (in 2012) overlaps NGPs (O’Toole et al., 2017). NGPs are not considered as microorganisms intended for human use so far. In Europe, the European Pharmacopoeia includes a class of products intended to prevent or treat diseases as medicinal products since 2019, which need to be registered under the rules for newly developed drugs (Sionek et al., 2023). This means that before application of NGPs, there should be a series of clinical trials (phase 1–3) to determine safety, dose range, side effects, and benefits. Hence, before *A. muciniphila* can be used in clinical treatment, there are still issues that require to be determined by further researches to ensure the safety and efficacy of treatment, and to obtain the approval for human-use.

## 8 Conclusion

*A. muciniphila* is a current star in the research field of next-generation probiotic because it colonizes in the mucus layer, a niche close to host cells, where it plays crucial role in gut homeostasis, exhibiting beneficial effects on several pathologies. *A. muciniphila* could produce small metabolites and mediators, affect microbial diversity and protect gut barrier integrity, thus exerting beneficial impacts on the gut and regulating a series of diseases including the metabolic, cardiovascular, infectious, and neurological diseases. The protective roles of *A. muciniphila* were reported for different infectious diseases including oral infection, respiratory infection, gut infection and systematic infection involving different pathogens such as bacteria and virus. The protection mainly act through reducing pathogen burdens, inhibition of the infection symptoms, and decreasing inflammation responses. Although preliminary data of the novel probiotic in the infectious diseases were inspiring. Further studies are required to reveal the exact roles of *A. muciniphila* in these areas and confirm the safety of *A. muciniphila* treatment, in particular to compare the effects of live, pasteurized and critical components of *A. muciniphila*, to test the efficacy of the protection roles in human beings.

## Author contributions

LL: Conceptualization, Funding acquisition, Writing – original draft, Writing – review & editing. ML: Formal analysis, Writing – review & editing. YC: Supervision, Writing – review & editing. ZeY: Investigation, Writing – review & editing. PC: Data curation, Writing – review & editing. ZhY: Validation, Writing – review & editing. WC: Supervision, Writing – review & editing. WZ: Supervision, Writing – review & editing. ZW: Resources, Writing – review & editing. XG: Formal analysis, Investigation, Writing – review & editing. HS: Data curation, Supervision, Writing – review & editing. XW: Investigation, Visualization, Writing – review & editing.
